# Correlation between the gut microbiota characteristics of hosts with severe acute pancreatitis and secondary intra-abdominal infection

**DOI:** 10.3389/fmed.2024.1409409

**Published:** 2024-08-21

**Authors:** Lihui Wang, Weijun Zhang, Simin Dai, Yuan Gao, Cheng Zhu, Yuetian Yu

**Affiliations:** ^1^Department of Critical Care Medicine, Renji Hospital, Shanghai Jiao Tong University, School of Medicine, Shanghai, China; ^2^Department of Disease Prevention and Control, Ruijin Hospital, Shanghai Jiao Tong University, School of Medicine, Shanghai, China; ^3^Key Laboratory of Intelligent Pharmacy and Individualized Therapy, Zhejiang, China; ^4^Key Laboratory of Multiple Organ Failure (Zhejiang University), Ministry of Education, Zhejiang, China

**Keywords:** severe acute pancreatitis, intestinal microecology, intra-abdominal infection, metagenomic next-generation sequencing, intensive care unit

## Abstract

**Objective:**

The objective of the study is to investigate the changes in the composition of intestinal microecology in severe acute pancreatitis (SAP) patients with or without intra-abdominal infection and also to analyze the expression of antibiotic resistance genes to provide evidence for early warning of infectious diseases and the rational use of antibiotics.

**Methods:**

Twenty patients with SAP were enrolled in the study. According to whether the enrolled patients had a secondary intra-abdominal infection, they were divided into two groups, each consisting of 10 patients. Stool specimens were collected when the patients were admitted to the emergency intensive care unit (EICU), and nucleic acid extraction was performed. Next-generation gene sequencing was used to compare the differences in intestinal microflora diversity and drug resistance gene expression between the two groups.

**Results:**

The gut microbiota of patients in the infection group exhibited distribution on multiple clustered branches with some intra-group heterogeneity, and their flora diversity was compromised. The infected group showed an enrichment of various opportunistic bacteria in the gut microbiota, along with a high number of metabolic functions, stress functions to external signals, and genes associated with pathogenesis. Drug resistance genes were expressed in the gut microbiota of both groups, but their abundance was significantly lower in the non-infected group.

**Conclusion:**

The intestinal microbiota of patients in the infection group exhibited distribution on multiple clustered branches with some intra-group heterogeneity, and their flora diversity was compromised. Additionally, drug resistance genes were expressed in the gut microbiota of both groups, although their abundance was significantly lower in the non-infected group.

## 1 Introduction

The prevalence of secondary intra-abdominal infection in severe acute pancreatitis (SAP) is notably high, primarily due to pathogens originating from the host’s intestinal tract (1, 2). Over 2000 species of intestinal microbes, constituting the gut microbiota, represent the most complex and significant micro-ecosystem in the human body. Metagenomic sequencing reveals that microbes within the human body encode genes exceeding the human genome’s gene count by a factor of 150 (3, 4). The role of the host’s intestinal microecology diversity, resistance, and metabolomic changes in SAP secondary intra-abdominal infection has been increasingly recognized. However, data from further controlled studies remain incomplete, lacking a foundation for developing diagnostic prediction systems or precision treatments.

Over the past decade, rapid advancements in metagenomic next-generation sequencing (mNGS) technology have enabled medical professionals to analyze the composition, structure, diversity, and drug resistance of gut microbiota (5–7). This progress facilitates further investigation into the potential pathogenesis of infectious diseases (8–11). Furthermore, gut microbes are crucial for the stability of gut microecology (12, 13). They establish a stable symbiotic relationship with both intestinal mucosal immune cells and intestinal epithelial cells. In critically ill patients, alterations in gut microecology can disrupt the stable co-existence of gut flora, resulting in complications such as microbial homeostasis loss, bacterial translocation, and enterogenic sepsis (14).

Secondary intra-abdominal infection in SAP has a high prevalence and is associated with a poor prognosis, predominantly caused by *Enterobacteriaceae*. Changes in the intestinal microbiota and bacterial translocation are hypothesized to significantly contribute to the disease’s pathogenesis. Consequently, we conducted a prospective cohort study using metagenomic sequencing to examine the compositional changes in the intestinal microbiota of SAP patients, both infected and non-infected, to facilitate early detection of infectious diseases. Furthermore, we seek to identify the expression patterns of dominant antibiotic resistance genes to inform the judicious use of prophylactic antimicrobials in clinical settings.

## 2 Materials and methods

### 2.1 Study design and setting

This prospective cohort study was conducted at the emergency intensive care unit (EICU) of Ruijin Hospital from July 2023 to December 2023. The Ethics Committee of Shanghai Jiao Tong University School of Medicine approved the study (No. 2023-RES-184), and the study was conducted in accordance with the Declaration of Helsinki (as revised in 2013). Written informed consent was obtained from all the enrolled patients. The design, implementation, and presentation of this study were strictly in accordance with the STROBE statement (15).

### 2.2 Participants

Adult patients admitted to the EICU and diagnosed with SAP were enrolled in our study. Patients were excluded if they met any of the following criteria: (1) upon admission to the EICU, in addition to SAP, concurrent one or more infectious diseases; (2) with a history of broad-spectrum antibiotic use 90 days before EICU admission; and (3) those who developed infectious diseases other than secondary abdominal infection during the course of hospital stay such as hospital-acquired pneumonia (HAP). They were categorized into two groups: the secondary intra-abdominal infection group and the non-infection group, each comprising 10 patients. Fresh stool samples were collected immediately following the definitive SAP diagnosis and admitted to the EICU. Both groups of patients did not receive antimicrobial therapy before sample collection.

### 2.3 Disease definition

According to the guidelines from the World Society of Emergency Surgery (WSES), acute pancreatitis can be diagnosed by meeting any two of the following three criteria: (1) sudden onset of acute upper abdominal pain radiating to the waist or back; (2) serum amylase and/or lipase levels in blood samples at least three times higher than the normal upper limit; (3) typical pancreatic lesions can be detected by contrast-enhanced computed tomography (CT) scan or magnetic resonance imaging (MRI) of the upper abdomen. If the patient has organ (one or more) dysfunction lasting more than 48 h after adequate fluid resuscitation, then they are diagnosed with SAP (16).

Secondary intra-abdominal infection may be suspected (16) when patients with acute pancreatitis meet one of the following criteria: (1) newly onset (body temperature ≥ 38.5°C) or persistent fever; (2) elevated levels of inflammatory markers [white blood cell count (WBC), neutrophil count, procalcitonin (PCT), or C-reactive protein (CRP)]; and (3) clinical symptoms continue to deteriorate, leading to secondary organ dysfunction; and at the same time, combined with any of the following: (1) the presence of gas bubbles in necrotic pancreatic tissue can be detected by an abdominal enhanced CT scan and (2) positive culture results can be obtained from percutaneous fine needle aspiration of the abdomen.

### 2.4 Nucleic acid extraction, library construction, and sequencing

The QIAGEN QIAamp PowerFecal DNA Kit facilitates the extraction of whole-genomic DNA from fecal samples. The purity and integrity of the extracted DNA are assessed via agarose gel electrophoresis, while its concentration is accurately measured using the Qubit 2.0 system. Samples exhibiting no diffuse degradation of nucleic acids and containing more than 10 ng of nucleic acid proceed to library construction. Following quality control, libraries are pooled based on their effective concentration and the desired data output volume. Sequencing is then performed using Illumina PE150, generating a minimum of 60 million reads per sample.

### 2.5 Gene prediction

Gene prediction is conducted using MetaGeneMark, followed by deduplication of the predicted genes to construct a gene catalog. This catalog forms the foundation for a comprehensive analysis of the clean data from each sample, yielding abundance information for the gene catalog in each instance.

### 2.6 Species annotation, diversity analysis, and differential microbiota analysis

The gene catalog, derived from gene prediction, is aligned with the MicroNR database and integrated with gene abundance data to generate species abundance profiles across different taxonomic levels. Species and functional abundance profiles are analyzed using methods such as abundance clustering, PCA, NMDS dimensionality reduction, and ANOSIM. Composition differences among samples, in terms of species and functions, are examined through LEfSe multivariate statistical analysis and comparative metabolic pathway analysis.

### 2.7 Annotation of antibiotic resistance genes

Utilizing the gene catalog and antibiotic resistance gene database for annotation provides sequential insights into the abundance, species attribution, and resistance mechanisms of antibiotic resistance genes.

### 2.8 Statistical analysis

Categorical variables are presented as counts (*n*) and percentages (%). They were compared using the chi-square (*χ*^2^) test or Fisher’s exact test when the sample size was less than five. Non-normally distributed data were compared using the Wilcoxon rank-sum test and are reported as medians [with an interquartile range (IQR)]. A two-sided *p*-value of <0.05 was considered statistically significant.

## 3 Results

### 3.1 Patient characteristics

The study included 20 patients with SAP, with 10 patients in each group, all of whom had biliary pancreatitis. Every enrolled patient had an intestinal feeding tube using a gastroscope for enteral nutrition within 48 h of admission to the EICU, and none of them received probiotic therapy during the course of the disease. Both groups of patients did not receive antimicrobial therapy during hospitalization before sample collection. After fecal sample collection, all patients with biliary pancreatitis were treated with a combination of third-generation cephalosporins (ceftazidime) and metronidazole. The median time for a secondary peritoneal infection to occur is 11 days. None of the 10 patients in the infected group developed intestinal fistula or necrosis. The main pathogens causing infection are *Escherichia coli* and *Klebsiella pneumoniae*, with three cases each. The baseline characteristics, inflammatory markers, intra-abdominal pressure, and prognosis indicators of the two groups of patients are listed in Table 1.

**Table 1:** Baseline characteristics of the study population.

Characteristic	Infection group	Non-infection group	*p*-value
	(*n* = 10)	(*n* = 10)	
Male gender, *n* (%)	5 (50.0)	5 (50.0)	1.00
Age, median [IQR]	41 [33, 56]	43 [31, 53]	0.32
Body mass index, median [IQR]	22.9 [18.3, 24.6]	22.1 [19.5, 25.3]	0.19
SOFA score	8 [5, 12]	7 [5, 11]	0.12
APACHE II score	15 [10, 21]	10 [6, 18]	0.03
Scr, median [IQR], umol/L	74 [57, 117]	68 [52,108]	0.17
Hb, median [IQR], g/L	85 [73, 99]	80 [71,103]	0.31
WBC, median [IQR], 10*^9^count/L	12.9 [7.3, 14.7]	10.7 [6.3,13.5]	0.02
PLT, median [IQR], 10*^9^count/L	118 [78, 225]	121 [98,262]	0.11
ALT, median [IQR], U/L	45 [17, 67]	41 [21,59]	0.21
AST, median [IQR], U/L	42 [18, 57]	47 [20, 59]	0.12
ALB, median [IQR], g/L	33.1 [28.7, 38.1]	34.3 [29.8, 36.1]	0.32
GLU, median [IQR], mmol/L	8.5 [6.4, 12.9]	9.6 [4.8, 12.7]	0.23
PCT, median [IQR], ng/mL	7.8 [5.2, 10.2]	5.8 [3.1, 7.6]	0.02
CRP, median [IQR], mg/L	156.3 [76.3, 205.9]	79.5 [55.2, 98.4]	0.04
IAP, median [IQR], mmHg	15 [7, 24]	14 [8, 23]	0.32
30-day mortality, *n* (%)	3 (30)	1 (10)	0.58

ALB, Albumin; ALT, Alanine aminotransferase; AST, Aspartate aminotransferase; APACHE II, acute physiology and chronic health evaluation II; CRP, C-reactive protein; GLU, Glucose; Hb, hemoglobin; IAP, intra-abdominal pressure; PCT, procalcitonin; PLT, platelet; Scr, serum creatinine; SOFA, sequential organ failure assessment; WBC, White blood cells.

### 3.2 Analysis of microbial community structure

**Figure 1:**
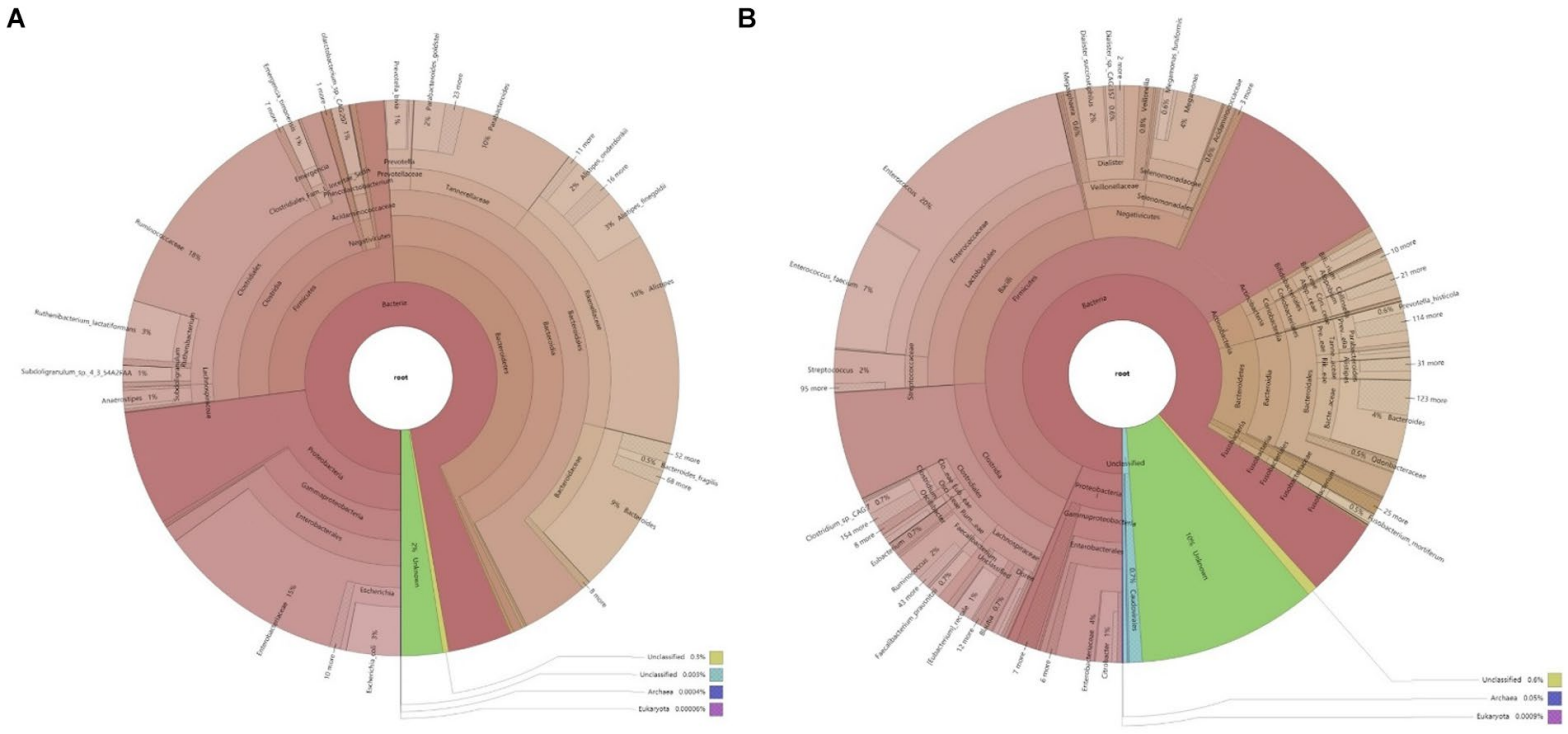
Krona chart of microbial composition in the samples. **(A)** Microbial composition of fecal samples from infected patients; **(B)** Microbial composition of fecal samples from non-infected patients.

Clustering analysis was performed using the Bray–Curtis distance metric, constructing a sample clustering tree to examine similarities. Clustering results across taxonomic levels were combined with species’ relative abundances in each sample for visualization. The analysis revealed that bacteria, with their higher abundance, are the predominant microbial communities in the feces of both infected and uninfected patients. The primary differences in microbial taxa between groups predominantly arise from bacteria (Figure 1). In the non-infected group, patients 1–7 exhibited highly similar microbial community structures, whereas patients 8–10 were more akin to those in the infected group. Furthermore, a 2-week follow-up revealed that these three patients developed peritoneal infections. Samples from the infected group spanned multiple clustering branches, suggesting a degree of intra-group heterogeneity.

**Figure 2:**
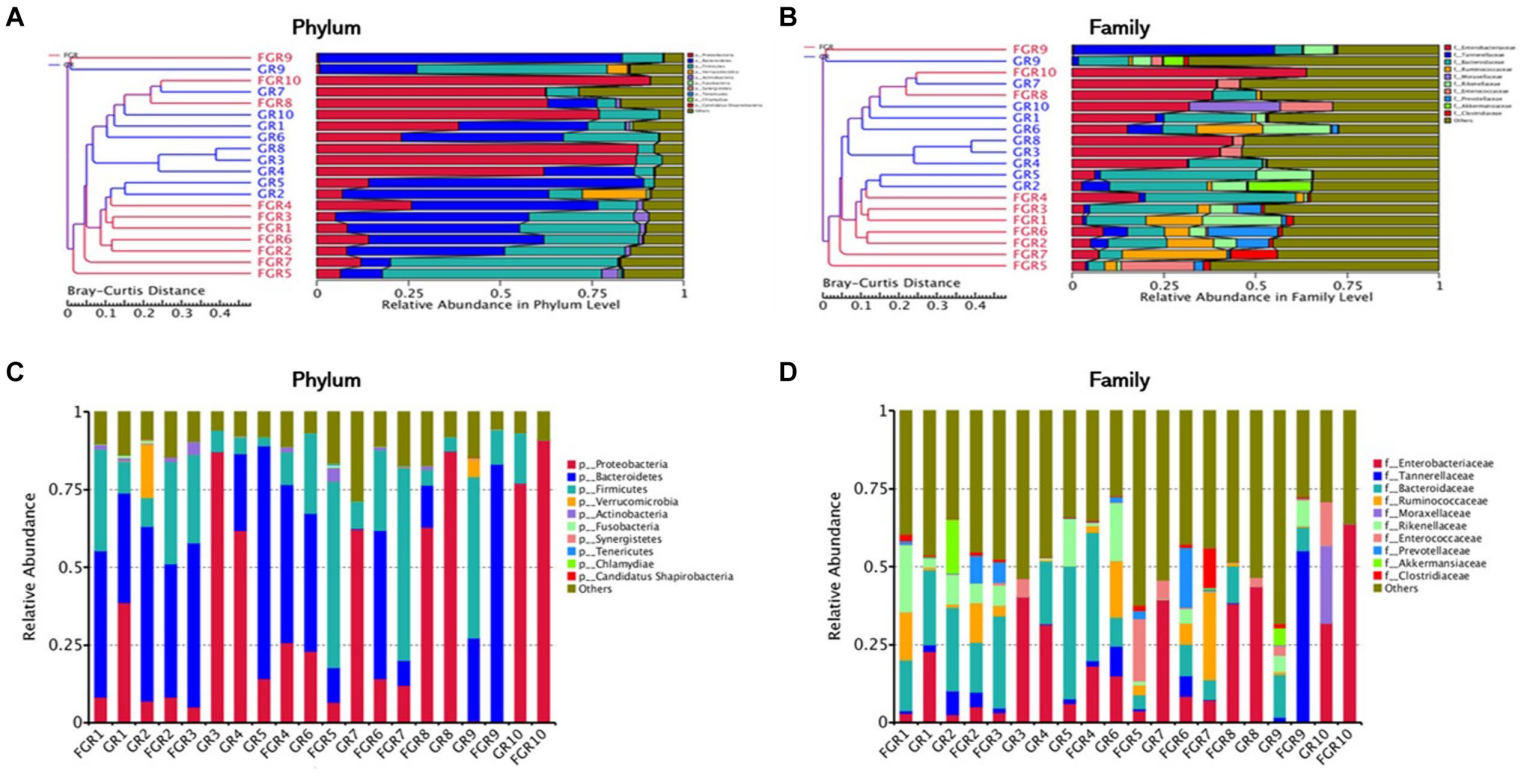
Cluster analysis of samples. **(A)** Cluster analysis of samples based on Bray–Curtis distance metric at the phylum level; **(B)** cluster analysis of samples based on Bray–Curtis distance metric at the family level; **(C)** stacked chart of the top 10 microbial compositions at the phylum level; **(D)** stacked chart of the top 10 microbial compositions at the family level.

The analysis of the microbial structure within and across samples reveals distinct differences between the infected and non-infected groups. In the non-infected group, the predominant bacterial phyla in feces are *Firmicutes* and *Bacteroidetes*, with a lower abundance of *Proteobacteria*. This microbial composition mirrors that of healthy individuals. The microbial composition of the infected group varies among individuals. Comparing the phylum-level abundance between groups reveals a higher abundance of *Proteobacteria* in the feces of infected patients. At the family level, *Enterobacteriaceae* are more abundant in the infected group than in the non-infected group, comprising various opportunistic pathogens. Genus-level analysis indicates a higher abundance of *Klebsiella pneumoniae* in the feces of some infected patients (Figure 2). *Klebsiella pneumoniae*, an opportunistic pathogen, typically exists in low abundance within the intestinal microecology in a non-pathogenic symbiotic form. The increased abundance of *Klebsiella pneumoniae* in the infected group suggests alterations in the intestinal microbial composition, disruption of the normal steady state, and a potential shift from a non-pathogenic symbiotic state to a pathogenic state.

### 3.3 Diversity analysis

**Figure 3:**
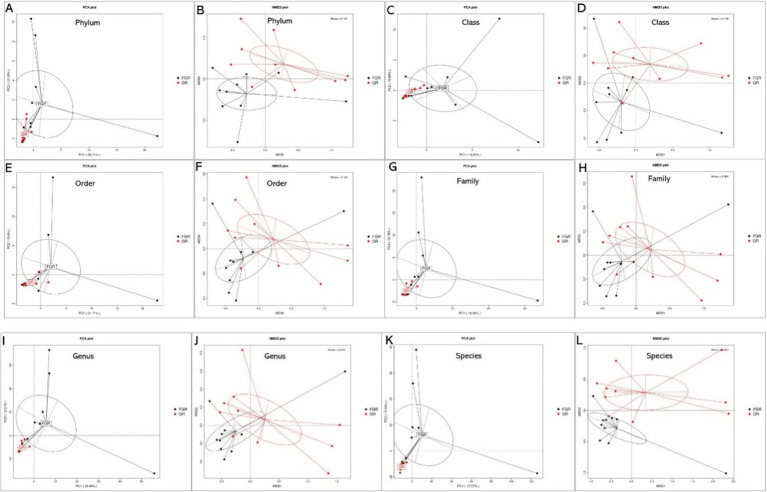
PCA and NMDS results of species at different levels between the two groups. **(A)** Phylum-level PCA; **(B)** phylum-level NMDS; **(C)** class-level PCA; **(D)** class-level NMDS; **(E)** order-level PCA; **(F)** order-level NMDS; **(G)** family-level PCA; **(H)** family-level NMDS; **(I)** genus-level PCA; **(J)** genus-level NMDS; **(K)** species-level PCA; **(L)** species-level NMDS. In PCA analyses, the *x*-axis and *y*-axis denote the first and second principal components, respectively, with the percentages reflecting the contribution of each principal component. Each sample is represented by a point in the figure, with samples from the same group depicted in identical colors. In NMDS analyses, points in the figure represent samples, with the distance between points within the same group reflecting sample repeatability. The closeness of samples within a group indicates the variation in hierarchical distance among group samples. The distance between points signifies the level of dissimilarity among samples, with samples from the same group shown in the same color. An NMDS stress value below 0.2 denotes a meaningful graphical analysis.

This study employed PCA and NMDS analyses to examine species abundance across various classification levels among samples and between infected and non-infected groups. Microbial species similarity is reflected by their proximity in PCA and NMDS plots. PCA and NMDS clustering analyses at the phylum level revealed variations in fecal microbiota composition between infected and non-infected groups, as illustrated in Figure 3.

### 3.4 Analysis of differential microbial communities

**Figure 4:**
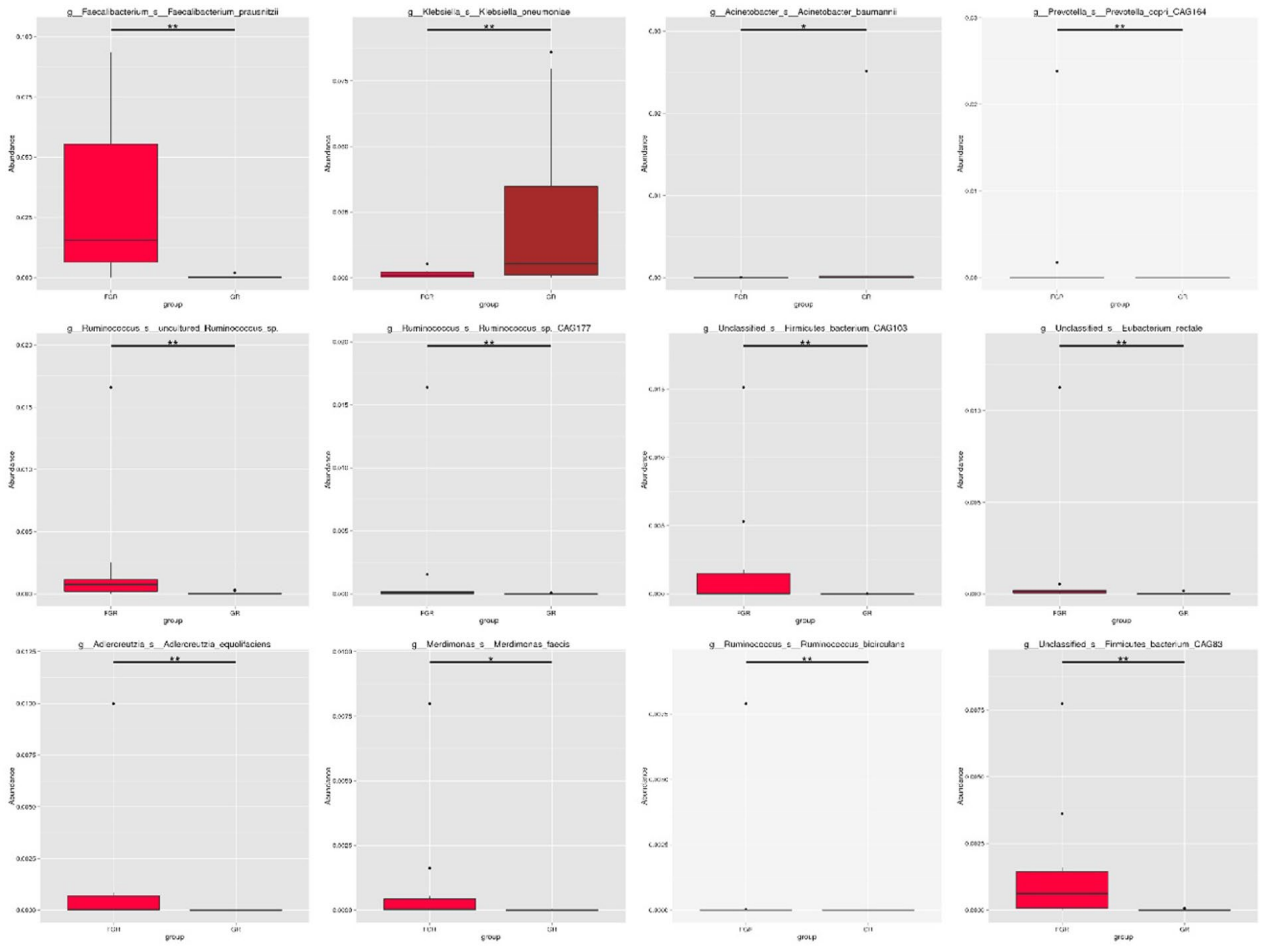
Box plot showing species with statistical differences at the species level. The horizontal axis denotes sample grouping, while the vertical axis indicates the relative abundance of the corresponding species. A horizontal line signifies the presence of statistical differences between two groups; its absence suggests no statistical difference exists for that species between the groups. *p < 0.05, **p < 0.01.

**Figure 5:**
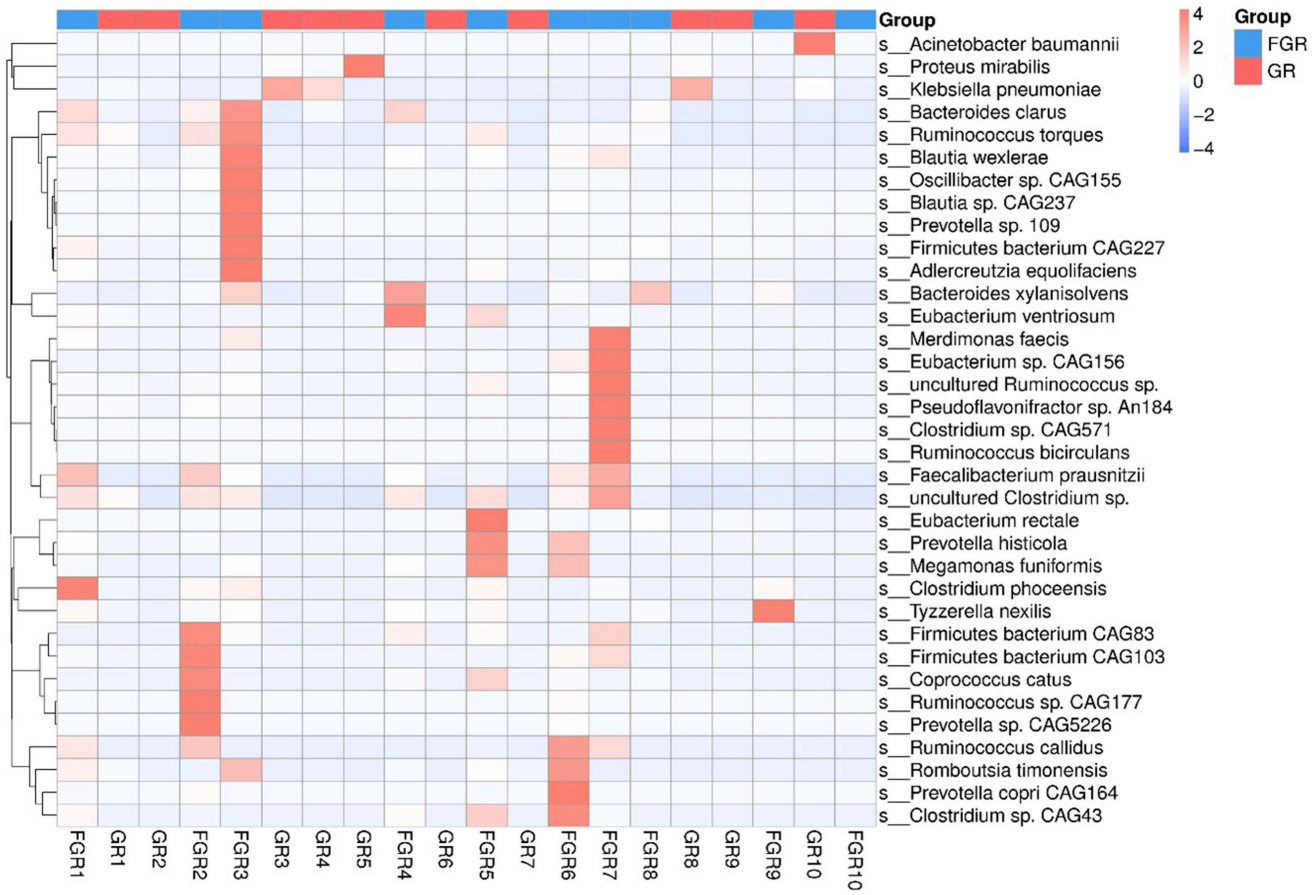
Heatmap of microbes detected in patients at the species level.

This study employed the Metastats method for statistical analysis of species abundance data, highlighting significant intergroup differences. It identified species with notable disparities and illustrated the abundance distribution using box plots. Figure 4 shows the 12 bacterial species exhibiting the most pronounced differences at the species level between the groups. This includes *Klebsiella pneumoniae* and *Actinobacter baumannii*, two conditionally pathogenic bacteria significantly more prevalent in the infected group. In the non-infected group, bacterial communities, including *Faecalibacterium*, *Ruminococcus*, and *Precotella*, which are involved in intestinal substance metabolism and short-chain fatty acid synthesis, showed higher abundance compared to the infected group. This finding suggests that in the infected group, the normal intestinal flora structure may be disrupted due to factors such as disease state and the use of antimicrobial drugs (Figure 5).

### 3.5 Functional gene structure

**Figure 6:**
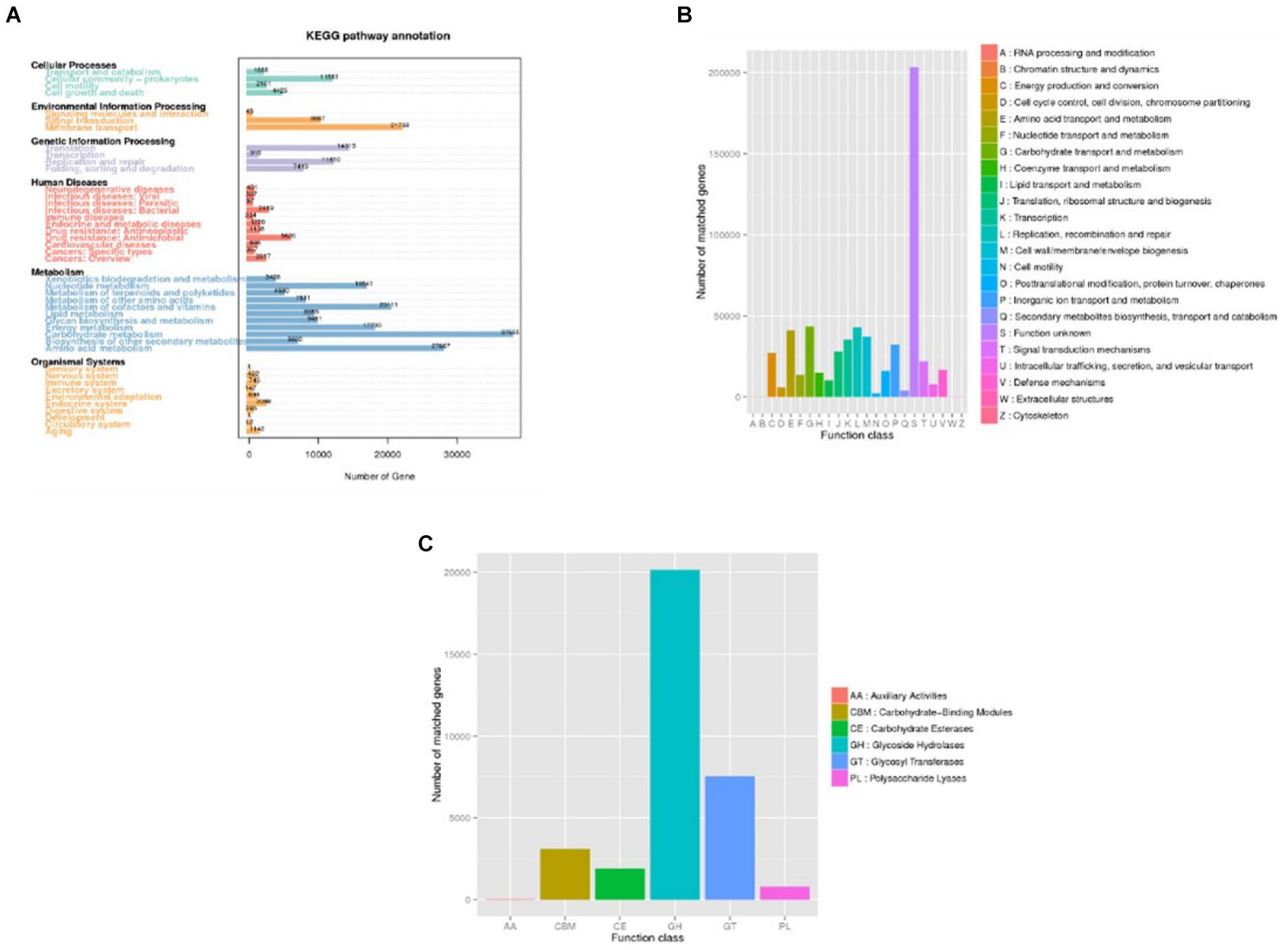
Distribution of annotated genes (Unigenes) across databases. **(A)** KEGG; **(B)** eggNOG. **(C)** CAZy. In panel **(A)**, the horizontal axis represents the number of annotated genes, while in panel **(B,C)** this role is assumed by the vertical axis. Conversely, the vertical axis in panel **(A)** and the horizontal axis in panel **(B,C)** depict the predicted levels of description, thereby illustrating the distribution of sample annotations across various functional categories.

Upon analyzing the functional genes across all specimens, it was observed that the fecal microbiota from the 20 patients contained a significant number of functional genes associated with metabolic processes (Figure 6), particularly those involved in carbohydrate metabolism. These findings support the role of the gut microbiome in facilitating the digestion and breakdown of nutrients.

By analyzing the functional gene types of each sample and group, differences in the microbial structure between the infection group and the non-infection group can be observed. Both KEGG and eggNOG annotations show that microbial metabolism, stress response to external signals, and pathogen-related genes are more abundant in patients from the infection group. ANOSIM indicates that the differences between groups are greater than those within samples, confirming the existence of disparities between the infection group and the non-infection group. Level 1 analysis of eggNOG reveals significant differences in gene functions between the two groups.

### 3.6 Resistance gene analysis

**Figure 7:**
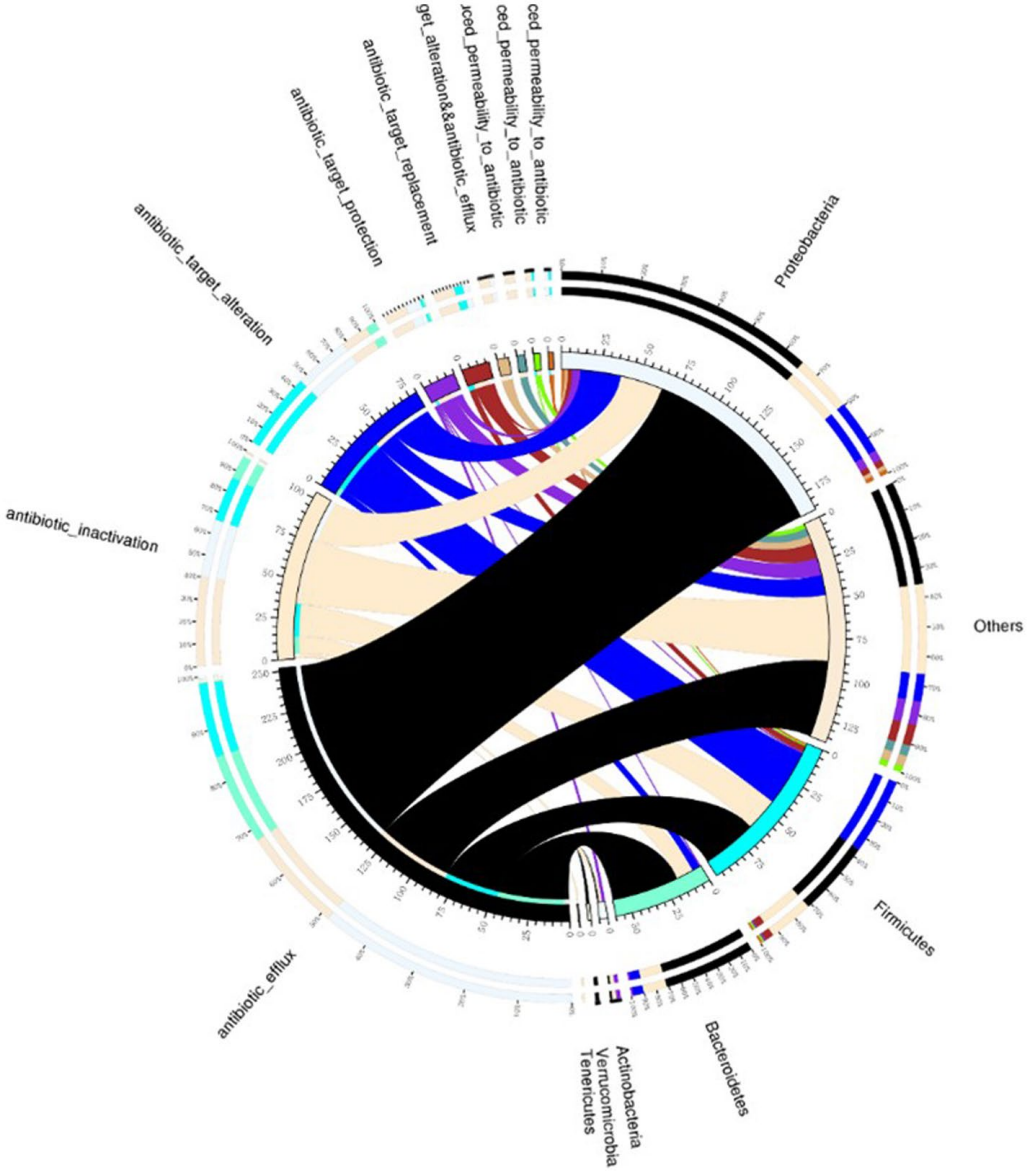
Venn diagram illustrating resistance mechanisms and species across all samples. The right side of the Venn diagram displays species information at the phylum level, and the left side outlines resistance mechanisms. The large circle on the left shows the percentage of resistance genes in each species, sorted by resistance mechanism. In contrast, the right side depicts the proportion of resistance genes in each mechanism, linked to specific species. The color of each small circle represents the corresponding species and resistance mechanisms, with the scale indicating the number of resistance genes. The left side shows the total number of genes associated with a specific resistance mechanism, whereas the right side presents the overall count of resistance genes.

**Figure 8:**
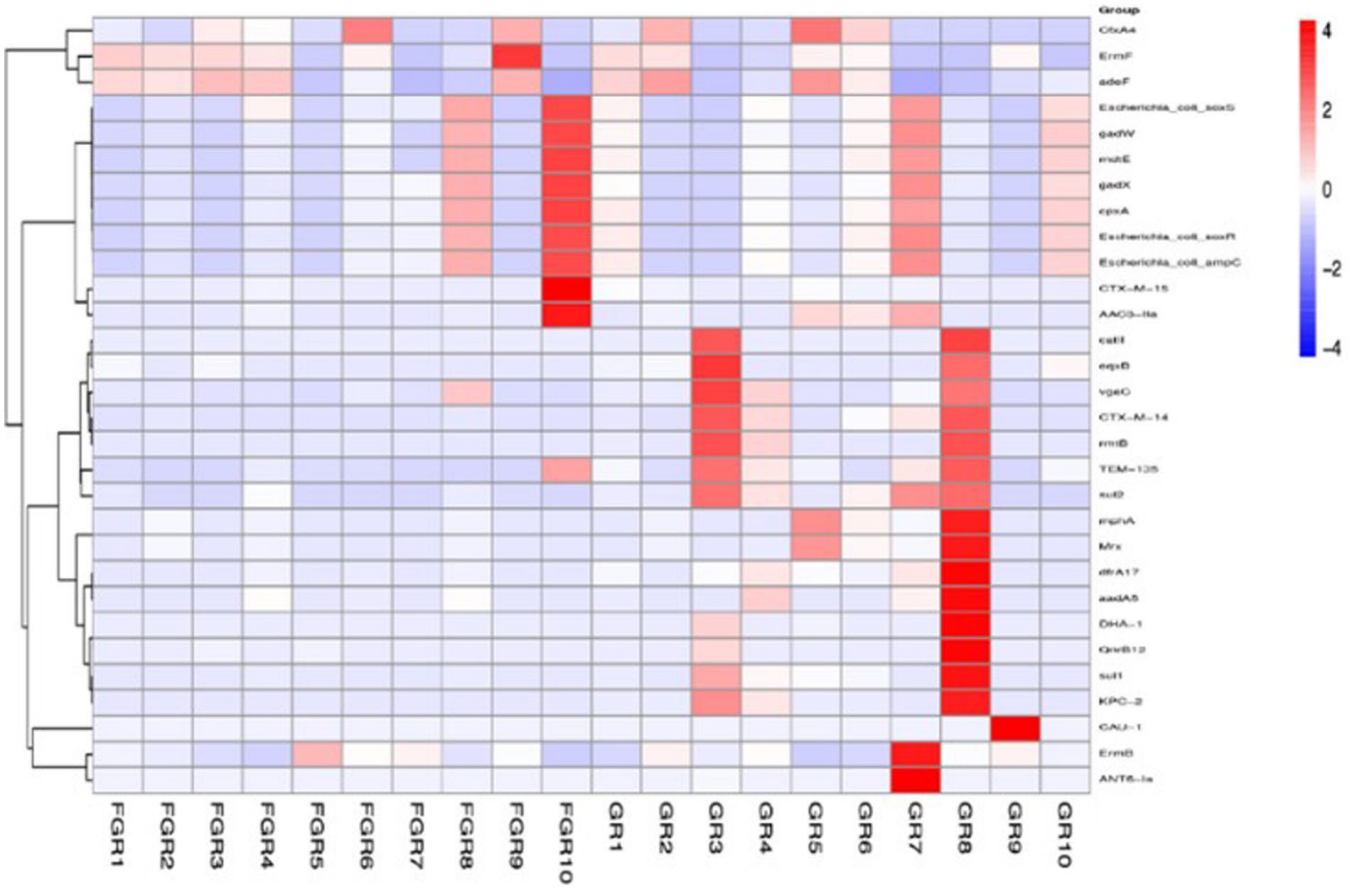
ARO distribution and abundance clustering heatmap. The horizontal axis represents the sample names, and the vertical axis on the right represents the names of the antibiotic resistance gene types (ARO).

Human intestinal microbiota frequently harbor antibiotic resistance genes. Antimicrobial drug usage has disrupted the stable co-existence of microbial species, impacting human health and ecological stability. Consequently, resistance gene research has garnered widespread attention. This study’s specimens contained a significant number of resistance genes (Figure 7). Focusing solely on the types of resistance genes, 222 types were common to both infected and non-infected groups. The non-infected group had a slightly higher number of unique resistance genes compared to the infected group. Despite no difference in gene types (ARO), a significant disparity in resistance gene abundance was observed between the groups. ANOSIM, based on resistance gene abundance, revealed a significant difference between the infected and non-infected groups. The heatmap (Figure 8) indicated an accumulation of resistance genes in the feces of infected patients, likely due to the selective pressure from antimicrobial drug use.

## 4 Discussion

We collected fecal samples from SAP patients with and without secondary intra-abdominal infection. Metagenomic deep sequencing analysis revealed that the gut microbiota of the infected group displayed distribution across multiple clustering branches, indicating intra-group heterogeneity and affected microbial diversity. The gut microbiome of the infected group was enriched with various opportunistic pathogens, exhibiting enhanced metabolic and stress response functions, as well as an increase in pathogenesis-related genes. Both groups showed antibiotic resistance gene expression in their gut microbiome. However, the non-infected group had significantly lower levels of these genes compared to the infected group.

The gut microbiota maintains host physiological balance and acts as a crucial barrier against pathogen invasion. It is essential for nutrition, energy metabolism, immune responses, and defending against infections (3). Research indicates that alterations in gut microecology significantly contribute to the development of inflammatory diseases, especially in the abdominal cavity. In SAP and similar conditions, a rapid release of inflammatory mediators can alter gut microbiota composition, degrade the colonic mucus layer, and increase intestinal mucosal permeability. Consequently, this leads to reduced colonization resistance, facilitating gut flora translocation, higher rates of enterogenic infections (e.g., *Enterobacteriaceae*, *Pseudomonadaceae*, and *Clostridium difficile*), and the emergence of metabolic diseases (4).

Colonization resistance refers to the gut microbiota’s role in preventing colonization by foreign bacterial pathogens (17, 18). As described in the 1960s, this phenomenon is primarily attributed to direct microbial inhibition (19). The abundant gut microbiota compete for scarce nutrients and epithelial cell adhesion sites, thus preventing overgrowth and potential pathogen invasion. Besides directly competing for nutrients and ecological niches, the gut microbiota indirectly combats invading pathogens by boosting the host’s gut immune defense (immune-mediated colonization resistance). Overall, these direct and indirect mechanisms synergistically prevent potential pathogen colonization and invasion, thereby inhibiting bacterial translocation and enterogenic infection (20, 21). The controversial use of prophylactic antimicrobial drugs in SAP patients can reduce the diversity of intestinal symbiotic bacteria and their direct inhibitory effects. Consequently, antimicrobial-resistant bacteria like vancomycin-resistant *Enterococci*, carbapenem-resistant *Enterobacteriaceae*, and *Clostridium difficile* can proliferate, occupy the mucosal surface, and cause bacterial translocation, severe enterogenic infections, intra-abdominal infections, and bloodstream invasion (22). This aligns with findings from the group with secondary infections in severe acute pancreatitis in this study, characterized by various conditional pathogens and a high abundance of resistance genes. Although the exact mechanisms of colonization resistance remain unclear, this study indicates that alterations in gut microecology diversity could be a contributing factor. A future research direction involves diminishing colonization of conditional pathogens, lowering the expression of resistance genes, and mitigating secondary infections through the restoration of gut microbiota integrity using probiotics (23).

In summary, our study indicates that severe acute pancreatitis secondary infections impact the diversity of intestinal microecological flora in patients. The intestines of these patients exhibit a rich presence of various opportunistic pathogens, enhanced metabolic and stress response functions to external signals, and an increased number of pathogenesis-related genes, along with a high expression of antibiotic resistance genes.

## Data Availability

The raw data supporting the conclusions of this article will be made available by the authors, without undue reservation.
